# Supplementary material for: Resolving the full spectrum of human genome variation using Linked-Reads
Source: Genome Res. 2019 Apr;29(4):635–45. doi: 10.1101/gr.234443.118 (PMC6442396; doi:10.1101/gr.234443.118)
Supplement: Supplemental Material [file supp_gr.234443.118_Supplemental_longranger-2.2.zip › longranger-2.2.tar.bz2!/lr2.2-public/lib/go/src/code.google.com/vitess/docs/user-guide/troubleshooting.html]

Vitess / Troubleshooting


Toggle navigation

- Overview
- Guides
- Reference
- Blog
- GitHub

- #### Overview

  - What is Vitess
  - Scaling MySQL with Vitess
  - Key Concepts
- #### Getting Started

  - Run Vitess on Kubernetes
    - Custom Docker Build
  - Run Vitess Locally
- #### User Guide

  - Introduction- Client Libraries- Backing Up Data- Reparenting
        - Schema Management
        - VSchema Guide
        - Schema Swap (Tutorial)
        - Sharding
          - Horizontal Sharding (Tutorial, automated)
          - Horizontal Sharding (Tutorial, manual)
          - Sharding in Kubernetes (Tutorial, automated)
          - Sharding in Kubernetes (Tutorial, manual)
        - Vitess Sequences
        - MySQL Server Protocol
        - Vitess and Replication
        - Update Stream
        - Row Based Replication
        - Topology Service
        - Transport Security Model
        - Launching
          - Scalability Philosophy
          - Production Planning
          - Server Configuration
          - 2PC Guide
          - Troubleshooting
        - Upgrading
- #### Reference Guides

  - Vitess API- vtctl Commands
- #### Other Resources

  - Presentations- Blog- Roadmap
- #### Contributing

  - Contributing to Vitess- GitHub Workflow- Code Reviews
- GitHub

# Troubleshooting

- #### Overview

  - What is Vitess
  - Scaling MySQL with Vitess
  - Key Concepts
- #### Getting Started

  - Run Vitess on Kubernetes
    - Custom Docker Build
  - Run Vitess Locally
- #### User Guide

  - Introduction- Client Libraries- Backing Up Data- Reparenting
        - Schema Management
        - VSchema Guide
        - Schema Swap (Tutorial)
        - Sharding
          - Horizontal Sharding (Tutorial, automated)
          - Horizontal Sharding (Tutorial, manual)
          - Sharding in Kubernetes (Tutorial, automated)
          - Sharding in Kubernetes (Tutorial, manual)
        - Vitess Sequences
        - MySQL Server Protocol
        - Vitess and Replication
        - Update Stream
        - Row Based Replication
        - Topology Service
        - Transport Security Model
        - Launching
          - Scalability Philosophy
          - Production Planning
          - Server Configuration
          - 2PC Guide
          - Troubleshooting
        - Upgrading
- #### Reference Guides

  - Vitess API- vtctl Commands
- #### Other Resources

  - Presentations- Blog- Roadmap
- #### Contributing

  - Contributing to Vitess- GitHub Workflow- Code Reviews

If there is a problem in the system, one or many alerts would typically fire. If a problem was found through means other than an alert, then the alert system needs to be iterated upon.

When an alert fires, you have the following sources of information to perform your investigation:

- Alert values
- Graphs
- Diagnostic URLs
- Log files

Below are a few possible scenarios.

### Elevated query latency on master

Diagnosis 1: Inspect the graphs to see if QPS has gone up. If yes, drill down on the more detailed QPS graphs to see which table, or user caused the increase. If a table is identified, look at /debug/queryz for queries on that table.

Action: Inform engineer about about toxic query. If it’s a specific user, you can stop their job or throttle them to keep the load manageable. As a last resort, blacklist query to allow the rest of the system to stay healthy.

Diagnosis 2: QPS did not go up, only latency did. Inspect the per-table latency graphs. If it’s a specific table, then it’s most likely a long-running low QPS query that’s skewing the numbers. Identify the culprit query and take necessary steps to get it optimized. Such queries usually do not cause outage. So, there may not be a need to take extreme measures.

Diagnosis 3: Latency seems to be up across the board. Inspect transaction latency. If this has gone up, then something is causing MySQL to run too many concurrent transactions which causes slow-down. See if there are any tx pool full errors. If there is an increase, the INFO logs will dump info about all transactions. From there, you should be able to if a specific sequence of statements is causing the problem. Once that is identified, find out the root cause. It could be network issues, or it could be a recent change in app behavior.

Diagnosis 4: No particular transaction seems to be the culprit. Nothing seems to have changed in any of the requests. Look at system variables to see if there are hardware faults. Is the disk latency too high? Are there memory parity errors? If so, you may have to failover to a new machine.

### Master starts up read-only

To prevent accidentally accepting writes, our default `my.cnf` settings
tell MySQL to always start up read-only. If the master MySQL gets restarted,
it will thus come back read-only until you intervene to confirm that it should
accept writes. You can use the SetReadWrite
command to do that.

However, usually if something unexpected happens to the master, it's better to
reparent to a different replica with EmergencyReparentShard. If you need to do planned maintenance on the master,
it's best to first reparent to another replica with PlannedReparentShard.

### Vitess sees the wrong tablet as master

If you do a failover manually (not through Vitess), you'll need to tell
Vitess which tablet corresponds to the new master MySQL. Until then,
writes will fail since they'll be routed to a read-only replica
(the old master). Use the TabletExternallyReparented
command to tell Vitess the new master tablet for a shard.

Tools like Orchestrator
can be configured to call this automatically when a failover occurs.
See our sample orchestrator.conf.json
for an example of this.

Contact: vitess@googlegroups.com  **·**  
Announcements  **·**  
© 2017 Vitess powered by Google Inc
